# Effect of conjugated linoleic acid supplementation on quality of life in rectal cancer patients undergoing preoperative Chemoradiotherapy

**DOI:** 10.12669/pjms.332.11925

**Published:** 2017

**Authors:** Elnaz Faramarzi, Reza Mahdavi, Mohammad Mohammad-zadeh, Behnam Nasirimotlagh, Sarvin Sanaie

**Affiliations:** 1 Elnaz Faramarzi, PhD. Assistant Professor of Nutrition, Liver and Gastrointestinal Disease Research Center, Tabriz University of Medical Sciences, Tabriz, Iran; 2 Reza Mahdavi, PhD. Professor of Nutrition, Nutritional Research Center, Tabriz University of Medical Sciences, Tabriz, Iran; 3 Mohammad Mohammad-zadeh, Associate Professor of Radiotherapy, Dept. of Radiotherapy, Shahid Madani Hospital, Tabriz University of Medical Sciences, Tabriz, Iran; 4 Behnam Nasirimotlagh, Dept. of Radiotherapy, Shahid Madani Hospital, Tabriz University of Medical Sciences, Tabriz, Iran; 5 Sarvin Sanaie, MD, PhD. Assistant Professor of Nutrition, Tuberculosis and Lung disease Research Center, Tabriz University of Medical Sciences, Tabriz, Iran

**Keywords:** Chemoradiotherapy, Conjugated linoleic acid, Rectal cancer, Quality of life

## Abstract

**Objectives:**

This study set out with the aim of evaluating the effect of conjugated linoleic acid (CLA) supplementation on quality of life in rectal cancer patients undergoing to preoperative chemoradiotherapy.

**Methods:**

In this study, 33 volunteer patients with rectal cancer treated with preoperative chemoradiotherapy were allocated in the CLA (n=16) and the placebo groups (n=17). The CLA group and placebo groups received 3 gr CLA/d and 4 placebo capsules for 6 weeks respectively. Before and after intervention, quality of life of patients was assessed by EORTC QLQ-C30.

**Results:**

At the end of study, the mean scores of physical function, role function, and cognitive function enhanced significantly in the CLA group while reduced remarkably in the placebo group. Symptom scales improved in the CLA group at the end of study. Comparison of changes in fatigue, pain and diarrhea scores were statistically significant between two study groups (P<0.05). When we compared the global health status scores between two groups, significant changes were observed (P<0.001).

**Conclusion:**

It appears that CLA may be helpful in rectal cancer patients by improving global quality of life. Although, other clinical trials with large sample size are needed to achieve more precise results.

## INTRODUCTION

Colorectal cancer is one of the most common cancers in the world, and it is the third most common cancer in both men and women.^1^ The main treatments for rectal cancer include surgery and preoperative chemoradiotherapy (CRT) or postoperative CRT. Multiple anti-cancer treatment increase risk of malnutrition in these patients.^2^ The suggested mechanism for inducing of malnutrition by radiotherapy in these patients is that exposure to clinically relevant doses of ionizing radiation can active cytokine cascades which stimulate several biological reactions at the cell and tissue levels. Indeed, it has been reported inflammatory factors (e.g. TNF-α, IL-1β, IL-6, etc) have an important role in metabolic disorders, muscle wasting, and development of symptoms such as anorexia, nausea, fatigue, and pain which affect nutrition status and quality of life of cancer patients.^3^

Thus, it is assumed that new adjuvant anti-inflammatory therapeutic approaches as a complementary therapy may be helpful in preventing of malnutrition and cachexia by down regulate inflammatory signaling pathways.^4^ Therefore, preventing of incidence of side effects of cancer treatments by using new adjuvant anti-inflammatory therapeutic approaches can improve nutrition status and quality of life of cancer patients. In this regard, the findings of previous studies have documented that there is significant association between nutrition status and quality of life in cancer patients.^3^,^5^ Taking into account that anti-inflammatory and anticancer effects of conjugated linoleic acid (CLA) has been noted by previous studies.^6^-^9^ Conjugated linoleic acids (CLAs) are fatty acids which consist of heterogeneous group of positional and geometric isomers of linoleic acid. Foods originated from ruminant animals (such as dairy products and meats) are the rich source of CLA.^10^

Despite the beneficial effect of CLA on inflammation and malnutrition, as far as we know, no studies have been found that evaluated effect of CLA supplementation on quality of life in cancer patients.

Considering the importance of supportive care in cancer patients and the important role of inflammation in incidence of symptoms which influence quality of life of cancer patients and anti inflammatory effect of CLA, we examined the effect of CLA supplementation on quality of life in rectal cancer patients treated with preoperative chemoradiotherapy.

## METHODS

Thirty-three volunteer patients with rectal cancer were chosen from the radiotherapy center of Imam Hospital in Tabriz. Written informed consent was obtained from all patients. This randomized clinical trial (RCT) was approved by Ethics Committee of Tabriz University of Medical Sciences and registered as the RCT study (IRCT: 201012041197N9).

Inclusion criteria were rectal cancer patients in stage II or III (based on TNM staging; Tumor-Node-Metastasis), who were candidate to treat neoadjuvant chemoradiotherapy. Treatment protocol is described in details in our previous study.^8^ Exclusion criteria were: history of any other cancer, radiotherapy and chemotherapy treatments; underweight (BMI<18.5 kg/m2); vitamin and mineral supplementation within the last month; diabetes; and liver, renal or endocrine dysfunction. The random sequence was performed by computer and the random sequence was kept in a remote secure location. An independent third party who was not involved in the study allocated patients in two groups. Patients and those who were involved in enrolling participants and administering interventions were blind to group assignments. Patients were allocated in the CLA (n=16) and the placebo groups (n=17). CLA group received four 1000 mg capsules (providing 3g CLA) three times/d (1 capsule at breakfast and dinner; two capsules at lunch) and placebo group(n=17) received four placebo capsules for 6 weeks. Placebo capsules contained sunflower oil and their appearance was similar to the CLA capsules. The CLA capsules provided from Tonalin, Natural factor, Canada which included two active isomers 18:2 c9, t11 and 18:2 t10, c12 in a 50/50 ratio. According to the previous study, we decided to use 3 g/d of CLA in this study.^7^ For inducing optimal serum levels of CLA, patients receive supplements one week prior to commencing chemoradiotherapy (loading period) and continued everyday up to the end of treatment.

Patients were checked weekly for any side effects of supplementation. The patients that consumed less than 90% of the planned number of capsules were not included in the analysis. Before and after intervention, quality of life of patients was assessed by European Organization for Research and Treatment of Cancer Quality of Life Questionnaire version.3.0 (EORTC QLQ-C30), downloaded from the EORTC website.^11^

The EORTC QLQ-C30 includes Global Health Status\QoL, Functional scales and Symptom scale/ items. Functional scales contain physical, role, emotional, cognitive, and social functioning. In Global health status \QoL and Functional subclasses, high scores indicate a high QoL and a high level of healthy functioning respectively. Symptom scale/ items include fatigue, nausea and vomiting, pain, dyspnoea, insomnia, appetite loss, constipation, diarrhea and financial difficulties; higher scores show increased severity of symptom.^12^,^13^

EORTC QLQ-C30 is a valid and reliable tool to evaluate quality of life cancer patients. This questionnaire has been translated to different languages. The Persian questionnaire was downloaded from the EORTC website which had been validated previously.^14^

The scoring of different parts of QLQ-C30 was calculated on the guideline of QLQ-C30 scoring. We accessed to this guideline by downloading from the EORTC website (Table-I), albeit the permission for using this guideline has been already obtained.

**Table-I:** Scoring the QLQ-C30.

	*Number of items*	*Item range*	*Item numbers*
**Global health status / QoL**			
Global health status/QoL	2	6	29,30
**Functional scales**			
Physical functioning	5	3	1 to 5
Role functioning	2	3	6,7
Emotional functioning	4	3	21 to 24
Cognitive functioning	2	3	20,25
Social functioning	2	3	26,27
**Symptom scales / items**			
Fatigue	3	3	10,12,18
Nausea and vomiting	2	3	14,15
Pain	2	3	9,19
Dyspnoea	1	3	8
Insomnia	1	3	11
Appetite loss	1	3	13
Constipation	1	3	16
Diarrhoea	1	3	17
Financial difficulties	1	3	28

The scoring of QLQ-C30 was performed as described below:

Raw Score = RS= (I_1_+I_2_+…+I_n_)/n

### Linear transformation

-Functional scales: S={1-(RS-1)/range)}*100

-Symptom scale/items: S={(RS-1)/range)}*100

-Global health status/QL: S={(RS-1)/range)}*100

### Statistical Analysis

The data were analyzed using Statistical Package for the Social Sciences (SPSS, version 11.5, Chicago, IL). The normality of data was assessed by Kolmogorov Smirnov test and the descriptive tests. The independent *t* test, χ^2^ test, paired-t-test, Signed test, and Mann-Whitney U test were used for data analysis. Effects of confounding factors was adjusted by analysis of covariance test (baseline values of variables). The result of this study is significant at the *P* value of less than 0.05.

Finally, analysis performed on thirty-one patients (CLA group, n=15; Placebo group, n=16). In the CLA group, one patient consumed less than 90% of the planed number of capsules because of forgetting to take the capsules regularly and in the placebo group, one patient was hospitalized and withdrew from the study.

## RESULTS

Comparison of baseline characteristics of patients showed no significant differences between two groups (Table-II). The mean scores of physical function, role function, and cognitive function changed insignificantly in the CLA group while reduced remarkably in the placebo group are shown in Table-III.

**Table-II:** Baseline characteristics of study groups.

	*CLA group(n=15)*	*Placebo group(n=16)*	P*
Age (year)	62.4±15.6	58.05±16.4	0.44
Gender n(%)			0.75
Male	8(53.3.8%)	10(62.5%)	
Female	7(46.7%)	6(37.5%)	
Stage rectal cancer n(%)**			0.78
II	6(40%)	5 (31.3%)	
III	9(60%)	11 (68.3%)	
BMI(kg/m^2^)	25.35±4.26	24.85±3.39	0.72

BMI=Body mass index,

* independent-t-test;

** based on TNM staging,¶ χ^2^test

**Table-III:** Comparison of scores for selected functional and symptom scales before and after intervention in CLA and placebo groups.

	*CLA group (n=15)*				*Placebo group (n=16)*			

	*Before*	*After*	*p*	*Change*	*Before*	*After*	*p*	*Change*
***Functional Scales***								
Physical function	80.51±24.56	85.12±20.21	*0.58	4.61(-13.2-22.43)	77.22±28.20	63.88±38.39	*0.13	13.33(-33.44-6.77)
Role function	88.46±18.49	89.74±21.01	**0.81	1.28(-10.67-13.24)	83.33±23.57	70.83±40.27	*0.3	-12.5(-38.09-13.09)
Emotional function	63.49±37.96	24.35±29.94	**0.02	-39.1(-76.58- -1.62)	91.66±12.81	27.08±28	*<0.001	-64.58(-85.65—43.50)
Cognitive function	90.21±18.06	98.71±4.6	**0.16	7.69(-3.65-19.03)	91.02±17.5	90.27±18.66	**0.08	-9.72(-21.19-1.75)
***Symptom scales/item***								
Fatigue	16.23±19.57	¶7.6±17.20	*0.11	-8.54(-23.59-6.50)	16.60±27	39.81±38.33	*0.02	23.14(1.97-44.31)
Nausea and vomiting	5.12±14.24	3.84±9.98	*0.85	-1.28(-12.5-9.94)	5.5±14.79	13.88±28.21	*0.46	8.33(-11.6—28.27)
Pain	30.76±25.31	¶12.82±21.68	0.04	-17.94(-37.43-1.52)	23.61±31.34	51.38±36.55	*0.01	27.77(6.92-48.63)
Dyspnea	10.25±21.01	7.69±19.95	*0.56	-2.56(-12.5-7.37)	2.77±9.62	8.33±20.71	*0.31	5.55(-6.67-17.78)
Insomnia	20.51±32.02	17.94±29.23	*0.59	-2.56(23.46-18.33)	11.11±21.71	36.11±43.71	*0.08	25(-3.73-53.74)
Appetite loss	10.25±21.01	10.25±21.01	-	-(-16.44-16.44)	19.44±38.81	27.77±31.24	*0.67	8.33(-25.6-42.27)
Constipation	15.38±29.23	2.56±9.24	*0.13	-12.82(-32.17-6.53)	19.44±30.01	8.33±20.71	*0.19	-11.1(-29.91-7.68)
Diarrhea	25.6±30.89	¶7.69±19.97	*0.0.6	-17.94(-37.43-1.53)	16.66±22.47	47.22±36.12	*0.01	30.55(9.45-51.65)

CLA, conjugated linoleic acid;a Mean value±standard deviation,

* P, comparison within group by paired-test;

** P, comparison within group by signed test,

¶ significantly different from the placebo group (P<0.05) by Mann Whitney U test.

Only significant changes were observed in scores of emotional functioning in both groups as compared with baselines (P<0.05). Symptom scales improved in the CLA group at the end of intervention. Comparison of changes in fatigue, pain and diarrhea scores were statistically significant between two study groups (P<0.05).

The changes of quality of life scores between study groups are shown in Table-IV. After intervention, the mean score of global health status increased significantly in the CLA group (P<0.001) and reduced in the placebo group (P=0.02) as compared with the beginning of the study. When we compared the global health status scores between two groups, significant changes were observed (P<0.001). CLA supplementation significantly increased functional scales scores.

**Table-IV:** Comparison of global health status, functional scales and symptom scales before and after intervention in CLA and placebo groups.

	*CLA group (n=15)*	*Placebo group (n=16)*	P*
***Global Health status***			
Before	70±14.363	73.71±19.19	
After	83.33±10.91	55.12±32.90	
**P(Before-After)	<0.001	0.02	
¶Change	12.22(5.51-20.12)	-17.36(-0.39- -34.32)	<0.001
***Functional Scales***			
Before	76.75±17.27	83.33±17.17	
After	86.15±12.99	72.22±28.24	
**P(Before-After)	0.11	0.17	
Change¶	9.40(-2.51-21.31)	-11.11(-27.97-5.75)	0.03
***Symptom, Scales***			
§Before	14.52±11.24	14.50±17.71	
§After	7.69±11.62	30.96±20.57	
§§P(Before-After)	0.14	0.01	
¶ Change	-7.49(-17.26-2.27)	15.90(3.29-28.52)	0.001

CLA, conjugated linoleic acid, change (differences of baseline and after intervention),

* P, comparison between group by independent t-test;

** P comparison within group by paired t –test;

§§ P comparison within group by Sign test, ANCOVA was used to adjust baseline values of variables;

¶ mean value (95% confidence interval);

§ Mean value±standard deviation.

After intervention, no significant changes in symptom scale scores found in the CLA group. Although, comparison of symptom scales between two groups revealed that there is a significant (P=0.001) difference (-7.49 vs 15.90).

## DISCUSSION

Nowadays, quality of life of cancer patients become one of the important issues in cancer patients’ treatment protocol. Several factors such as stage of disease, side effects of anti cancer treatments, depression and inflammation due to cancer or cancer treatments influence quality of life cancer patients. The results of previous studies indicated quality of cancer patients decreased significantly during chemotherapy/radiotherapy.^15^-^17^

In reviewing the literature, no study has been found that evaluated effect of CLA supplementation on quality of life in cancer patients. Thus, the findings of the present study were compared with studies that evaluated the effect of different supplementations (e.g. omega 3 fatty acids) on quality of life in cancer patients.

Lai et al observed body weight and quality of life scores of cachectic patients with head and neck or gastrointestinal cancer who received celecoxib (COX-2 inhibitors) increased significantly in comparison with the placebo group.^18^ Furthermore, other researchers reported that high dense energy and protein supplement contain n-3 fatty acid improved body weight, lean tissue, and quality of life patients with advanced pancreatic cancer.^19^

In another study, Maccio et al found that in patients with advanced gynecological cancer combination of L-carnitine+celecoxib+antioxidants (alpha lipoic acid and carboxycysteine)+Megestrol acetat (MA) was more effective than MA (alone) in improving global quality of life patients.^20^ Another results of their study were that inflammatory factors (IL-6, TNF-α, CRP) reduced significantly in the combination treatment group while these variables did not change in the MA group. They concluded that observed improving in quality of life patients were associated with decreasing inflammatory factors.

Considering the important role of inflammation in occurrence of symptoms due to cancer or cancer treatments, it can be assumed that the improvement of quality of life of patients in the present study may be ascribed to anti-inflammatory properties of CLA. This hypothesis was supported by the results of our previous study that 3gr/d CLA supplementation for 6 weeks in patients with rectal cancer treated with chemoradiothearpy improved inflammatory factors.^8^

After intervention, the average of functional scales scores enhanced in the CLA group and decreased in the placebo group as compared with the beginning of the study. In comparison with the placebo group, CLA significantly increased functional scale scores.

Functional scales contain physical, role, emotional, cognitive, and social functioning. High scores in functional scales indicate a high level of healthy functioning. It has been reported that nutritional status of patients with cancer influence quality of life.^3^ In this regard, Ravasco et al observed dietary counseling in colorectal cancer patients who received radiotherapy had better quality of life function scores corresponding their nutritional status and intake.^17^ Findings of another research showed that home parentral nutrition in cancer patients had positive effect on quality of life function scores by correcting or preventing malnutrition.^21^ Moreover, it has been reported using nutritional supplements with anti inflammatory effects can improve quality of life of cancer patients by reducing incidence of symptoms of eating problems.^22^ In line with this, Mahdavi et al found that CLA supplementation in rectal cancer patients improved nutritional status, symptoms of eating problems and dietary intake. They noted observed positive effects of CLA supplementation in cancer patients can be ascribed to anti-inflammatory effects of CLA.^9^ Therefore, positive effect of different nutritional interventions on nutritional status and symptoms of patients can lead to increase physical function, physiologic state and social well being.

At the end of the study, symptom scale scores increased in the placebo group. These results are consistence with those of another study which reported the mean scores of symptoms (such as diarrhea, fatigue, and appetite loss) significantly increased compared with the pretreatment scores.^16^ Also, the findings of another study showed that loss of appetite, nausea/vomiting and diarrhea enhanced significantly in patients with uterine cancer treated with radiotherapy.^15^ In comparison with the placebo group, CLA supplementation resulted in significant reduction of scores of symptom scales in the supplemented group. Similar to our results, beta glucan supplementation reduced significantly score of symptom scales in breast cancer undergoing chemotherapy.^23^

Therefore, the observed beneficial effect of CLA supplementation on occurrence of symptoms of eating problems and dietary intake of cancer patients can be attributed to anti-inflammatory effect of CLA which have been reported by some animal studies [24,25] and clinical trials.^6^-^8^

As far as we know this study is the first study which examined the effect of CLA supplementation on quality of life cancer patients. It is the strengths of this study. The limitation of this study is small sample size.

In conclusion, the results of our research showed that CLA supplementation improved global quality of life, functional scales and decreased score of symptom scales in comparison with the placebo group. It appears that CLA may be helpful in rectal cancer patients by improving global quality of life. Although, other clinical trials with large sample size are needed to achieve more precise results.

## References

[1] Torre LA, Bray F, Siegel RL, Ferlay J, Lortet-Tieulent J, Jemal A (2015). Global cancer statistics 2012. Cancer J Clin.

[2] Van Cutsem E, Arends J (2005). The causes and consequences of cancer-associated malnutrition. Euro J Oncol Nurs.

[3] Ravasco P, Monteiro-Grillo I, Vidal PM, Camilo ME (2004). Cancer:disease and nutrition are key determinants of patients'quality of life. Support Care Cancer.

[4] Argiles JM, Busquets S, Lopez-Soriano FJ (2011). Anti-inflammatory therapies in cancer cachexia. Euro J Pharmacol.

[5] Jager-Wittenaar H, Dijkstra PU, Vissink A, van der Laan BF, van Oort RP, Roodenburg JL (2011). Malnutrition and quality of life in patients treated for oral or oropharyngeal cancer. Head Neck.

[6] Bassaganya-Riera J, Hontecillas R (2010). Dietary conjugated linoleic acid and n-3 polyunsaturated fatty acids in inflammatory bowel disease. Curr Opin Clin Nutr Metab Care.

[7] Song H, Grant I, Rotondo D, Mohede I, Sattar N, Heys S, Wahle K (2005). Effect of CLA supplementation on immune function in young healthy volunteers. Euro J Clin Nutr.

[8] Mohammadzadeh M, Faramarzi E, Mahdavi R, Nasirimotlagh B, Jafarabadi MA (2013). Effect of conjugated linoleic Acid supplementation on inflammatory factors and matrix metalloproteinase enzymes in rectal cancer patients undergoing chemoradiotherapy. Integrative Cancer Therapies.

[9] Mahdavi R, Faramarzi E, Mohammad-Zadeh M, Nasirimotlagh B, Ghaemmaghami SJ (2013). Effects of conjugated linoleic acid supplementation on nutritional status, symptoms of eating problems and dietary intake in rectal cancer patients undergoing chemoradiotherapy. Curr Topics Nutraceutical Res.

[10] Chin S, Liu W, Storkson J, Ha Y, Pariza M (1992). Dietary sources of conjugated dienoic isomers of linoleic acid, a newly recognized class of anticarcinogens. J Food Composition Analysis.

[11] EORTC (2012). European Organisation for Research and Treatment of Cancer Quality of Life Questionnaire (EORTC QLQ-C30).

[12] Aaronson NK, Ahmedzai S, Bergman B, Bullinger M, Cull A, Duez NJ (1993). The European Organization for Research and Treatment of Cancer QLQ-C30:a quality-of-life instrument for use in international clinical trials in oncology. J Natl Cancer Inst.

[13] Fayers P, Aaronson N, Bjordal K, Groenveld M, Curran D, Bottomley A (2001). The EORTC QLQ-C30 Scoring Manual Published by:European Organisation for Research and Treatment of Cancer.

[14] Safaee A, Moghim Dehkordi B (2007). Validation study of a quality of life (QOL) questionnaire for use in Iran. Asian Pacific J Cancer Prevent.

[15] Ahlberg K, Ekman T, Gaston-Johansson F (2005). The experience of fatigue, other symptoms and global quality of life during radiotherapy for uterine cancer. Int J Nurs Stud.

[16] Guren MG, Dueland S, Skovlund E, Fossa SD, Poulsen JP, Tveit KM (2003). Quality of life during radiotherapy for rectal cancer. Euro J Cancer.

[17] Ravasco P, Monteiro-Grillo I, Vidal PM, Camilo ME (2005). Dietary counseling improves patient outcomes:a prospective, randomized, controlled trial in colorectal cancer patients undergoing radiotherapy. J Clin Oncol.

[18] Lai V, George J, Richey L, Kim HJ, Cannon T, Shores C (2008). Results of a pilot study of the effects of celecoxib on cancer cachexia in patients with cancer of the head, neck, and gastrointestinal tract. Head Neck.

[19] Fearon K, Von Meyenfeldt M, Moses A, Van Geenen R, Roy A, Gouma D (2003). Effect of a protein and energy dense N-3 fatty acid enriched oral supplement on loss of weight and lean tissue in cancer cachexia:a randomised double blind trial. Gut.

[20] MacciòA Madeddu C, Gramignano G, Mulas C, Floris C, Sanna E (2012). A randomized phase III clinical trial of a combined treatment for cachexia in patients with gynecological cancers:Evaluating the impact on metabolic and inflammatory profiles and quality of life. Gynecol Oncol.

[21] Culine S, Chambrier C, Tadmouri A, Senesse P, Seys P, Radji A (2014). Home parenteral nutrition improves quality of life and nutritional status in patients with cancer:a French observational multicentre study. Support Care Cancer.

[22] Laviano A, Seelaender M, Sanchez-Lara K, Gioulbasanis I, Molfino A, Rossi Fanelli F (2011). Beyond anorexia-cachexia. Nutrition and modulation of cancer patients' metabolism:supplementary, complementary or alternative anti-neoplastic therapy?. Euro J Pharmacol.

[23] Ostadrahimi A, Esfahani A, Asghari Jafarabadi M, Eivazi Ziaei J, Movassaghpourakbari A, Farrin N (2014). Effect of Beta glucan on quality of life in women with breast cancer undergoing chemotherapy:a randomized double-blind placebo-controlled clinical trial. Adv Pharm Bull.

[24] Yang M, Cook ME (2003). Dietary CLA decreased weight loss and extended survival following the onset of kidney failure in NZB/W F1 mice. Lipids.

[25] Changhua L, Jindong Y, Defa L, Lidan Z, Shiyan Q, Jianjun X (2005). Conjugated linoleic acid attenuates the production and gene expression of proinflammatory cytokines in weaned pigs challenged with lipopolysaccharide. J Nutr.

